# Cytotoxic effect of rosemary extract on gastric adenocarcinoma (AGS) and esophageal squamous cell carcinoma (KYSE30) cell lines 

**Published:** 2017

**Authors:** Neamat Karimi, Jalil Rashedi, Behroz Mahdavi Poor, Sepideh Arabi, Maryam Ghorbani, Nahideh Tahmasebpour, Mohammad Asgharzadeh

**Affiliations:** 1 *Department of Biochemistry, School of Medicine, Ardabil University of Medical Sciences, Ardabil, Iran*; 2 *Department of Laboratory Science, Faculty of Paramedicine, Tabriz University of Medical Sciences, Tabriz, Iran*; 3 *Department of Medical Parasitology, School of Medical Sciences, Tarbiat Modarres University, Tehran, Iran*; 4 *School of Medicine, Sari University of Medical Sciences, Sari, Iran*; 5 *Department of Biochemistry, Faculty of Medicine, Hamedan University of Medical Sciences, Hamedan, Iran*; 6 *Biotechnology Research Center, Faculty of Paramedicine, Tabriz University of Medical Sciences, Tabriz, Iran*

**Keywords:** Rosmarinus, adenocarcinoma, cytotoxicity, esophageal

## Abstract

**Aim:**

The present study was conducted to survey the potential cytotoxic influence of freeze-dried aqueous extract of its fruits on gastrointestinal cell lines, namely AGS (human gastric carcinoma) and KYSE30 (human esophageal squamous cell carcinoma.

**Background:**

Rosemary (*Rosmarinus officinalis*) is a wild medicinal plant shown to have anticancer activity. Carnosic and rosmarinic acids are compounds, obtained from it through several extraction methods.

**Methods:**

The aqueous extract of the fruits of *R.officinalis* was freeze-dried, and KYSE30 and AGS cancer cell lines were treated with crude extract. Cytotoxic effect of the extracts on the cell lines was examined using 3-(4, 5-Dimethylthiazol-2-yl)-2,5-diphenyltetrazolium bromide (MTT) and neutral red assay. Apoptotic cells were detected with ethidium bromide/acridine orange (EB/AO). Cell-cycle distributions were evaluated by flow cytometry.

**Results:**

IC50 values were 4.1, 1.8 and 1.3 mg/mL for AGS cell lines after 24, 48 and 72 hours by MTT assay, respectively, and 4.4, 2.1 and 1.1 mg/mL by neutral red assay, respectively. IC50 values for KYSE30 cell lines were 600, 180 and 150 mg/mL after 24, 48 and 72 hours by MTT assay, and 860, 270 and 230 mg/mL by neutral red. EB/AO staining increased in apoptotic cells. After 24 h of treatment at different concentrations, significant increases and decreases in population were shown at G2/M and G1 phases, respectively.

**Conclusion:**

The aqueous extract of the fruits of *R.officinalis* was freeze-dried, and KYSE30 and AGS cancer cell lines were treated with crude extract. Cytotoxic effect of the extracts on the cell lines was examined using 3-(4, 5-Dimethylthiazol-2-yl)-2,5-diphenyltetrazolium bromide (MTT) and neutral red assay. Apoptotic cells were detected with ethidium bromide/acridine orange (EB/AO). Cell-cycle distributions were evaluated by flow cytometry.

## Introduction

 Gastric cancer is an important public health challenge as the fourth most common cancer and the second cause of cancer mortality worldwide (1). This cancer is the most frequently diagnosed form of cancer in Iran (2). Both northern and northwestern parts of Iran are at high risk for gastric cancer (3). Esophageal cancer is one of the fatal cancers, constituting the eighth most common cancer in the world, and accounting for 4% of all cancers worldwide (2). It is the third most prevalent cancer of the gastrointestinal system (4). Worldwide, a high number of esophageal carcinoma patients die within a year of diagnosis and only 8-20% remain alive after 5 years (5). Plant-derived ingredients have been a notable source of several clinically useful anti-cancer agents (6). R. officinalis is a common household plant, which has a green branched bushy shrub dark green leaves and whitish-blue flowers. It grows wildly along the north and south coasts of the Mediterranean Sea (7-9). Caffeic acid and its derivatives such as Rosmarinic acid are the most important ingredients of rosemary. These compounds have antioxidant effect. The rosmarinic acid, phenolic compound, obtains one of its phenolic rings from phenylalanine (10). It is well demonstrated that excessive production of reactive oxygen species (ROS) may induce tissue damage and therefore play a critical role in the pathogenesis of various diseases. The epidemiological literature indicates that maintaining high intakes of antioxidants such as vitamin E and C, β-carotene and certain food components can help to protect against life threatening diseases such as cancer (11). Rosemary constituents have shown a variety of pharmacological activities for cancer chemo-prevention and therapy in vitro and in vivo (12-14). Although there are some studies on the cytotoxicity of this plant’s extract on several human cancers cell lines, it seems necessary to further explore the chemo-preventive properties of crude extract of rosemary (R. officinalis L) on Adenocarcinoma cell lines (AGS) and KYSE30; therefore, we investigated the in vitro cytotoxic activity of rosemary extract on these cell lines. 

## Methods

KYSE30 and AGS were purchased from the Pasteur Institute (Tehran, Iran). In order to provide the crude extract (12), two different lots of R. officinalis dry leaves were obtained from an herbs and spice store in Rasht, Iran. Collected seeds were dried and ground to yield a fine powder. A definite amount of powder was added to methanol and filtrated with filter paper. One part of the extract was kept away, and to separate the non-polar compounds, the rest of the extract was washed with solvents (dichloromethane, hexane, ethylacetate). Then, the solvents were removed with rotary evaporator to prepare the extract. Subsequently, the mixture was obtained and allowed to freeze at -70oC, then it was lyophilized with freeze dryer ALPHA 2-plus, and the remnant preserved at -20oC.

**Cell culture and treatment**

Both KYSE-30 and AGS cells were grown at 37oC on RPMI-1640 medium along with 10% FBS, streptomycin (100 µg/mL) and (penicillin 100 units/mL). Cell types were trypsinized and plated in 96 micro titer plates at a density of 1×104 cells per well, and incubated overnight. After attachment, the medium was removed and the cells were incubated for 24, 48 and 72 hours with a serum-free medium containing 10 mg/mL of the extract by ¼ serial dilutions. The toxicity effect of the extract was evaluated by MTT and neutral red assays.

**MTT assay**

The media were removed four hours before completion of the incubation time, 20µL of MTT (2.5 mg/mL), diluted 1:10 in serum free medium, was added to each well. Then, the media were removed and 200µL dimethylsulfoxide (DMSO) was added to each well after the plates were incubated for a further 4 h. The plates were shaken for 10 min at 100 rpm and absorbance was measured at 570 nm using a plate reader (Synergy HT, Biotek).

**Neutral red assay**

The media were aspirated from each well three hours prior to the end of the incubation time. Immediately, the neutral red solution (0.05%) was added to each well and incubated for 3 hours. At the end of incubation, the wells were washed with warm phosphate-buffered saline (PBS) (37oC) after the neutral red solution was removed. Finally, absorbance was measured at 540 nm on the plate reader after the addition of fixative solution (150 µL) to each well (15).

**EB/AO staining**

The cells were seeded in 96-well plates (density of 1×103 cells per well) and incubated overnight, then treated with 0.03-2×103 µg/mL of the extract after attachment. Apoptotic cells were stained after the plates were centrifuged for 5 min (129 g, 1000 rpm) at 4oC. Ten µL of EB/AO dye mix (100 µg/mL acridine orange and 100 µg/mL ethidium bromide mixed in PBS) was added to each well. An inverted fluorescence microscope was used to count the cells. All apoptotic cells were detected by condensed chromatin in perinuclear which was stained with ethidium bromide or acridine orange, and through the generation of apoptotic bodies. Necrotic cells were recognized via monotonous labeling of the cells with ethidium bromide (16). Cell-cycle analysis was conducted by flow cytometry after staining by DAPI method. (15). The PartecFloMax software was used to analyze the distribution of cells at different cell-cycle phases. 

**Statistical analysis**

The Sigma plot 11 software was used for calculation of concentration for IC50 value. The statistical significance of the results was assessed using Student’s t test; also, the Newman-Keuls test was applied for multiple comparisons. P<0.05 was considered as significant. 

## Results

**Growth inhibition**

**Figure 1:**
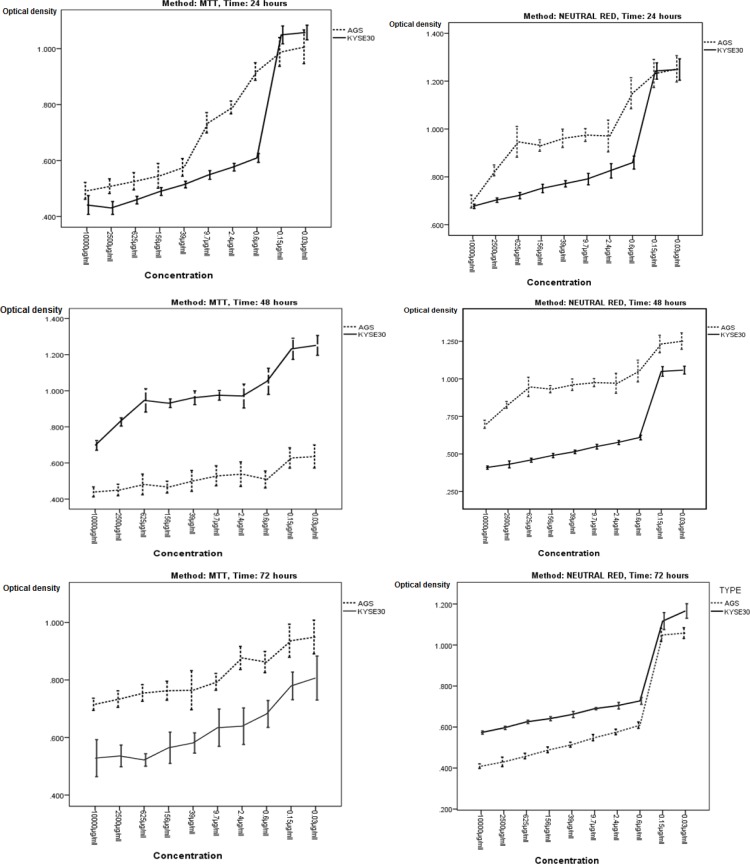
MTT and neutral red assay of cytotoxic activity of Rosemary on AGS and KYSE-30 cell lines in time- and dose-dependent manner.

Assessment of viability revealed that the crude extract of R. officinalits fruit had a cytotoxic impact on cancer cell lines following 24, 48 and 72 hours of incubation (Figure 1). The AGS cell line was more influenced by the cytotoxic effect of the extract. A decrease in cell viability was shown when assessed by the MTT and neutral Red. The IC50 values were 4.1, 1.8 and 1.3 mg/mL for AGS cell line after 24, 48 and 72 hours by MTT assay, respectively, and 4.4, 2.1 and 1.1 mg/mL for AGS cell line by neutral red assay, respectively. Also, IC50 values for KYSE30 cell line were 600, 180 and 150 mg/mL after 24, 48 and 72 hours by MTT assay, respectively, and 860, 270 and 200 mg/mL for KYSE30 cell line by neutral red, respectively.

**Figure 2:**
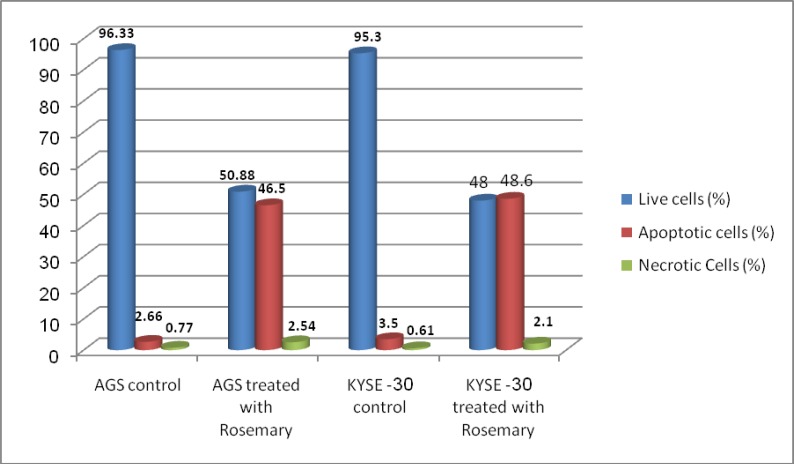
Apoptosis induction by Rosemary on AGS and KYSE-30 cell lines. Data are expressed as percentage of cells

**Figure 3:**
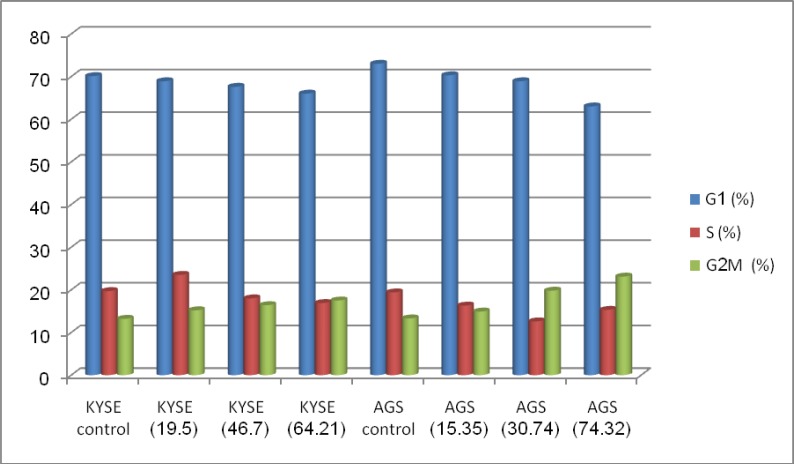
Effect of aqueous extract of Rosemary on cell-cycle distribution on AGS and KYSE-30 cell lines

EB/AO staining depicted the morphological characteristics of the treated cancer cells. The cells exhibited a series of morphological changes, including fragmentation and condensation of nucleus and chromatin, as well as formation of apoptotic bodies after incubation with 2-250 mg/mL of the extract for 24 hours. In contrast, control cells displayed a normal appearance. A significant increase of apoptotic cells was shown in cancer cells after treatment (figure 2) (P<0.001). To obtain information on cell cycle progression, the DNA content of the cancer cells treated with Rosmary during cell cycle phases was measured by flow cytometric cell-cycle analysis. For this purpose, the PartecFloMax software was used to calculate the percentage of cells in G1, S and G2/M phases. We showed a dose-dependent effect of Rosemary on cell cycle. The cells in the G2/M population increased compared to the controls after 24 h of Rosemary treatment at different concentrations. The rising cell population at the G2/M phase was seen along with a decline in cell population in the G1 phase of cell cycle (figure 3). The impact of Rosemary on cancer cells seems to be dose-dependent; i.e. the higher the dosage, the higher the increase in the G2/M population. 

## Discussion

The antioxidant characteristics of rosemary crude extract and its ingredients have been well evaluated. Most of these indicated antioxidant functions are based on cell types (13, 17). The results showed that the crude extract of R.officinalits fruit exerts cytotoxic effect on AGS and KYSE30 gastrointestinal cell lines. It was also observed that gastric cancer cells (AGS) were more sensitive to the cytotoxic effect of the extract. A number of studies have been performed to determine anti-proliferative and cytotoxic activity of Rosemary alcoholic extracts on different human cancerous cell lines, including HL60, K562, MDA, MCF-7, Hep-3B, K-562, DU-145, NCI-H82, MB-231, and PC-3. It has been reported that the IC50 value of the extract varies for different cell lines (18). In an experiment on 7,12-dimethylbenz[a]anthracene (DMBA) induced breast cancer in FNB/N type rats, it was revealed that DMBA resulted in higher expression of the genes involved in carcinogenesis, including c-myc and cyclin-D1, as well as activation of the NF-kB pathway. Moreover, DMBA induced chronic inflammation and ROS overproduction, causing oxidative damage of DNA (19). 

Application of Rosemary methanolic extract to rat skin inhibits covalent attachment of Benzopyrene to DNA. In addition, it was reported that Orsolic acid inhibits binding of DMBA and Benzopyrene to epidermal cell DNA, as well as NF-kB to the cell membrane. Orsolic acid of the Rosemary extract blocks the NF-kB pathway in cancerous cells, possibly via inhibition of P65 and NF-kB phosphorylation repressor and causing attenuation of COX-2, MMP-9, Cyclin D1, C-Jun and C-fos oncogenes. In addition, Rosemary contains Carnosol which has a similar activity and blocks the NF-kB (20). Rosemary leaves contain Carsonic acid which neutralizes reactive oxygen species, including hydroxyl and lipid peroxide radicals and thus protects biologic membranes against lipid peroxidation. It has been reported that Carsonic acid reduces lipid peroxidation by 88-100% under oxidative stress conditions. Orsolic acid could also reduce oxidative stress similarly (21). In addition, Rosemary could induce cell death in COLO 205 cells via mitochondrial and cell death receptor pathways which could be employed for cancer treatment and prophylaxis (12). 

This study demonstrated the cytotoxic effect of freeze-dried aqueous extract of rosemary fruit, by apoptosis, on gastrointestinal cancer cell lines. Gastric cancer cells (AGS) exhibited higher sensitivity. The mechanism for the inhibition was further investigated with cell cycle analysis. The results showed that Rosemary was able to induce G2/M cell cycle arrest in gastrointestinal cancer cell lines. This finding provides a new understanding of the cytotoxic effects of Rosemary on gastric and esophageal cancer cells.
